# On-Line Monitoring of Blind Fastener Installation Process

**DOI:** 10.3390/ma12071157

**Published:** 2019-04-10

**Authors:** Javier Camacho, Fernando Veiga, Mari Luz Penalva, Alberto Diez-Olivan, Lutz Deitert, Norberto López de Lacalle

**Affiliations:** 1 Tecnalia Research and Innovation, 20009 Donostia-San Sebastián, Spain; javier.camacho@tecnalia.com (J.C.); mariluz.penalva@tecnalia.com (M.L.P.); alberto.diez@tecnalia.com (A.D.-O.); 2 Airbus Operations GmbH, 28199 Bremen, Germany; lutz.deitert@airbus.com; 3 CFAA, Centre for Advanced Manufacturing for Aeronautics, Department of Mechanical Engineering, University of the Basque Country (UPV/EHU), 48013 Bilbao, Spain; norberto.lzlacalle@ehu.eus

**Keywords:** online process monitoring, process signal analysis, classification method for quality assessment, blind fasteners, *k*-means clustering, non-destructive inspection

## Abstract

Blind fasteners are of special interest for aircraft construction since they allow working on joints where only one side is accessible, as is the case in many common aerospace box-type structures, such as stabilizers and flaps. This paper aims to deliver an online monitoring method for the detection of incorrect installed blind fasteners. In this type of fastener, the back side of the assembly is not accessible, so monitoring the process installation is suitable as a system to assess the formed head at the back side with no access. The solution proposed consists of an on-line monitoring system that is based on sensor signals acquired during the installation. The signals are conveniently analyzed in order to provide an evaluation outcome on how the fastener was installed. This new method will help production to decrease/eliminate time and cost-intensive inspections and fasteners over installation in structures. The decrease of the number of installed fasteners will also contribute to weight savings and will reduce the use of resources.

## 1. Introduction

Among the large variety of fasteners used in aircraft components, assembly-blind fasteners are a specific type, which, for installation, just require access the front side of the assembly. This offers the chance of cheaper and easier automation solutions.

The fastening process is a challenging operation involving multiple knowledge fields (metallic materials, composite materials, classic mechanics, plastic deformation, machining and manufacturing, contact mechanics, tribology, and others). The scientific community has been involved in great efforts toward the understanding and modelling of the fastening process over the years [1,2] and also in developing methods for assessing the state of fastened joints [3,4].

Evaluating an installed blind fastener depends greatly, as shown in Table 1, on the examination of the formed head (sleeve and spindle) on the back side of the assembly. When blind fasteners are used in closed structures their evaluation after installation is not feasible without using time and cost-intensive equipment. Sometimes, no evaluation at all is possible. Quite often, these issues are solved by overcalculating the number of fasteners in order to meet safety requirements, though this leads to the increase of weight and production costs. Note that a commercial aircraft requires the installation of around 1,500,000–3,000,000 fasteners [5]. A reduction of 15% fuselage weight could be achieved by substituting riveting for alternative joining processes [6]. Under these conditions, it is clear that the potential benefits of blind fasteners are not currently being fully exploited. 

Therefore, a monitoring system for the installation of blind fasteners does not only involve an automated solution that alerts on faulty installations with a high degree of reliability and robustness, but also involves a solution to avoiding any direct inspection of the formed head (i.e., with no access to the assembly back side).

Inspection for the assessment of the installation of blind fasteners is an essential component to overcome to reduce aircraft manufacturing, maintenance, and operation costs. The lack of an effective method for the inspection of blind fasteners involves a certain degree of uncertainty. Due to such uncertainty, the manufacturing of aircrafts is penalized: i) directly, because blind fastened joints are currently designed with security factors that lead to the use of a bigger number of fasteners per joint, and this way airplanes are more expensive to manufacture, require longer manufacturing lead times, and are heavier, due to the increase in number of fasteners; and ii) indirectly, because the uncertainty entails a higher risk of failure and, thus, the use of blind fasteners is avoided in certain critical components, in favor of less suitable, but more confident, joining methods. In fact, “sensor-based process monitoring and control and automated quality inspection” was set as a key research challenge for improving the joining of dissimilar materials by Martinsen, Hu, and Carlson in Reference [7]. The industrial and scientific community are developing methods for taking advantage of the current on-line monitoring capabilities, resulting in large data quantities as the basis for the development of prognosis models (Diez et al. [8]). For example, Palasciano et al. [9] have developed a machine processing and sensing information-based model for abnormal energy consumption identification, caused not necessarily by failure conditions but by erroneous human-made inputs (dimensioning, established cutting conditions, and trajectory programming).

Although big efforts are being put into the inspection and maintenance of fastened joints, seeking for early detection of defects on the joints (cracks, corrosion, delamination, or others), there are no extended inspection techniques for assessing the quality of the installation itself. A review on current non-destructive methods for assessing fastened joints was performed by Thoppu et al. in Reference [10]. Aiming at assessing the appearance of corrosion in multi-layered structures, Le et al. [11] present a method for the inspection of the integrity of rivets themselves, which could be applicable to blind fasteners too. The method assesses the integrity of the nut, but it is not intended for assessing the quality of the installation (nor is it able to; an incorrect installation with a sound nut will not be detected by this method). Zhang et al. [12] developed a linear index (obtained after the analysis of the energy lost by an acoustic wave passing through a tight/loose bolt) to assess the tightening state of fastened joints, which could lead to early detection of degraded joints during service, but cannot assess the quality of the installation (an incorrect installation may lead to initial proper tightening of the joint). In [13], Camacho et al. propose a method for assessing the quality of the installation of blind rivets, based on one process parameter (cycle time) and one additional parameter to be measured after the installation: The time-of-flight of an ultrasonic pulse through the rivet nut. Saygin, Mohan, and Sarangapani [14] analyzed the fastening process using torque and angle signals and developed a Mahalanobis-Taguchi systembased approach for detecting the real grip length of each installed rivet, aiming at assessing if the grip rivet is used correctly at each installation. In Reference [15], a kernel density-based method is applied to the head diameter and the head height to automatically group data and remove outliers, and then identifies the classification of patterns for the corresponding torque vs rpm diagrams.

Current research work develops a method for assessing the installation of blind rivets, based on the analysis and processing of process signals measured online during the installation process. The main contribution of current work consists on a laboratory-stage method that has proved its potential for assessing the quality of the installation of blind rivets, which could be generalized for its industrial application.

## 2. Experimental Work

### 2.1. Riveting Process and Installation Assessment Criteria

**Figure 1:**
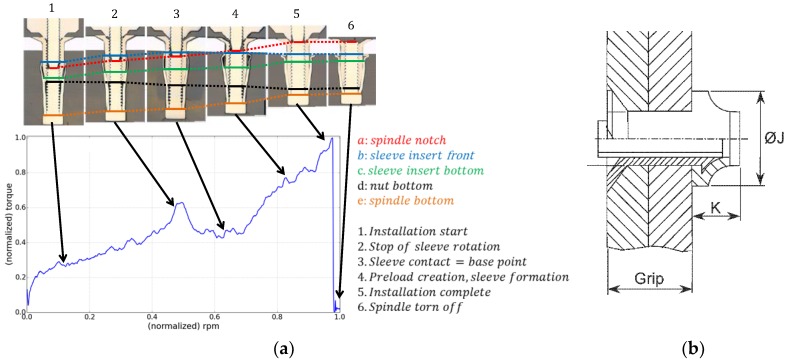
(**a**) Installation diagram and fasteners cross-sections for relevant diagram points; (**b**) quality-control parameters from the blind side: Formed head diameter (*J*) and formed head height (*K*) (source: Airbus).

Figure 1 shows a typical installation diagram (torque vs. rpm of the fastening spindle) for blind fasteners. In this research a method for assessing the quality of the resulting installation, based on the acquisition and analysis of the installation diagram, is presented and, thus, does not require additional operations other than the installation itself and the analysis of acquired data.

An installed blind fastener is classified as correctly installed (OK) or incorrectly installed (NOK), depending on the representative dimensions of its formed head, namely, the head diameter (*J*) and the head height (*K*), as shown in Figure 1. The “break off limit” was inspected from the accessible side of the joint and it was thus out of scope with current research. After installation, once the rivet was deformed and the blind rivet head was formed, the resulting blind head diameter *J* must have reached a minimum value. Accordingly, the resulting head height *K* must have been deformed enough as to be below its maximum allowed value. Currently, inspection of the blind formed head is not performed except for during initial machine installation and process set-up stages.

### 2.2. Generation of Failed Installation of Rivets 

Installing blind rivets incorrectly was not a straightforward operation. To that end, 3 different main approaches were tested:

- Inserting fasteners with incorrect grip-to-thickness relation (either higher or lower values).
- Misaligning the fastener by generating a 10° to 20° blind-side slope (which is out of tolerance).
- Modifying the process variables: Air pressure, initial preload, and initial nut position.

However, approaches ii and iii both proved to result in correct fastener installation according to both *J* and *K* criteria (see Table 2).

### 2.3. Experimental Setup

**Figure 2:**
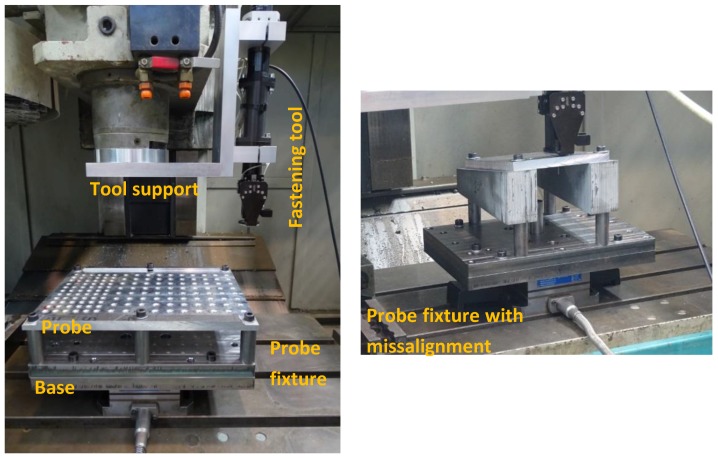
Experimental setup for rivet installation.

An experimental setup was designed for fastener installation analysis purposes, as shown in Figure 2. It was constituted by a 3-axis CNC milling center, in which a fastening tool-head was installed. Fasteners were installed into previously drilled probes, which were mounted onto a probe fixture. This whole assembly relied on a dynamometric plate capable of measuring forces along the 3 axes. In addition, the fastening head was capable or measuring the installation torque, spindle revolutions, air pressure and air flow. 

After the sample fastener installation generation and probe removal from the fixture, formed head diameter *J* and formed head height *K* were manually measured for all fasteners.

**Figure 3:**
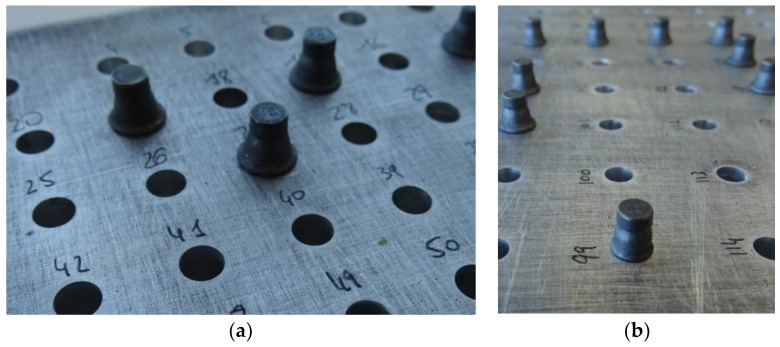
Sample installed fasteners: Formed heads on the blind side of the joint (**a**) correct rivets, (**b**) incorrect rivet (#99).

This way, a total of 108 sample fastener installations were produced. Table 2 describes their main characteristics, and Figure 3 shows examples of them. All fasteners used had the same type and dash: MBF2313-5 [16].

### 2.4. Data Preprocessing

**Figure 4:**
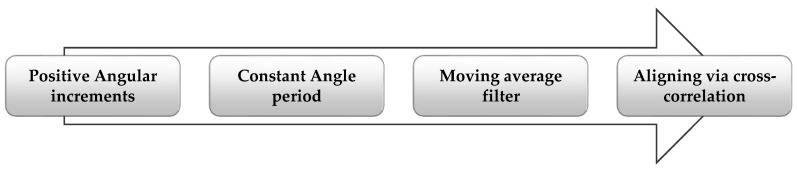
Preprocessing of acquired data: From time signals to normalized angular signals.

**Figure 5:**
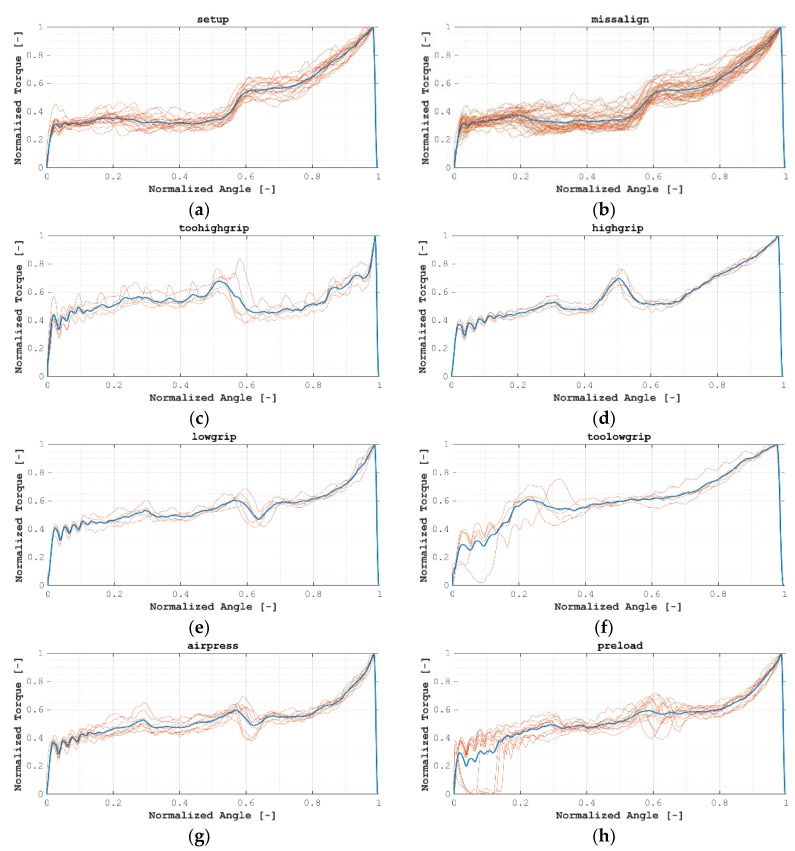

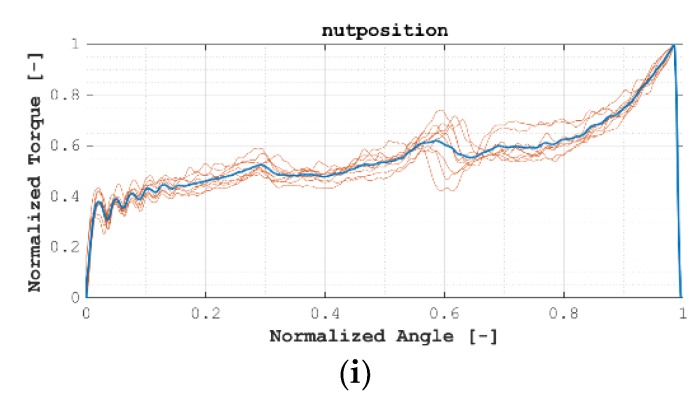
All normalized signals grouped by test case: (**a**) HTM01; (**b**) HTM02; (**c**) HTM03_A; (**d**) HTM03_B; (**e**) HTM03_C; (**f**) HTM03_D; (**g**) HTM04; (**h**) HTM05; (**i**) HTM06.

At the first stage of the analysis, acquired data had been pre-processed, aiming at data normalization and creating comparable data between each installation process. In this way, the process illustrated in Figure 4 was followed. At the first stage, data were switched from time domain to angular domain and was resampled at a constant angular period. A softening moving average filter was applied, and the resulting signal was normalized via cross-correlation to an artificial 0–1 normalized angular position and 0–1 torque values. Figure 5 shows all normalized signals.

## 3. Towards a Classification Method: Data Clustering

For the test samples it was possible to easily access the blind side of the joint and, thus, to measure directly *J* and *K* values and classify each installation as sound or faulty (OK or NOK). Consequently, having a direct classification of each test item, a supervised method may be expected as a classification algorithm, labeling an installation either as OK or NOK directly based on the prediction made by the classifier given by the normalized torque/normalized angle diagram of a newly tested installation.

Yet, it is believed that such classification may not be sensitive enough. It is hypothesized that the different normalized torque/normalized angle diagram may lead to OK installations and several different diagrams may lead to NOK installations. For instance, diagrams of installations of fasteners at the uppermost limit and the lowermost limit of the grip could present severe differences and still lead both to OK installations. 

That is, several failure mechanisms can lead to NOK installations, as seen in Table 1. Even for a single failure mode, it is considered that different angle-torque diagrams may lead to NOK installations.

Furthermore, a NOK installation means that the installation is unsuccessful; however, it can be due to different reasons (too low *J* or too high *K*, or even both too low *J* and too high *K*). In conclusion, the NOK label integrates all possible failure modes and, thus, it is considered as the union of subgroups. Clustering the data into groups is intended to identify such subgroups. 

The aim of this research is to study the applicability of unsupervised methods to establish the similarities and differences between OK and NOK installations in terms of normalized torque/normalized angle diagrams, without considering the *J*–*K* values. The latter values will be used to evaluate the achieved results.

For that reason, the use of unsupervised classification methods is suggested in current research; for instance, the *k*-means algorithm, one of the most popular clustering methods [17]. The *k*-means algorithm is an unsupervised clustering method which will group together similar samples into a predefined number of clusters, ‘*k*’. The similarity between two samples was measured as the Euclidean distance between them, considering each point in the normalized angle/normalized torque signal as a degree of freedom or coordinate. That is, each point of the curve was considered as an attribute. The *k*-means algorithm will seek to minimize the total distance of all riveted installations by assigning each installation to the closest cluster.

**Figure 6:**
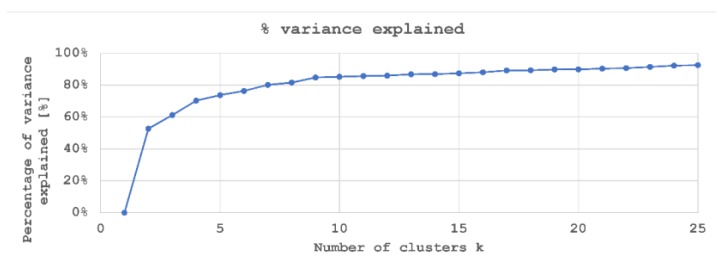
Elbow graph for k-means clustering.

This way, two separate analyses were performed, which consider different numbers of clusters:

- Grouping into 2 clusters, *k* = 2.
  Although, as explained, the NOK label was expected to gather several subgroups, being the aim to classify installation as either OK or NOK, the ability to obtain directly the classification from normalized torque signals is studied.
- Grouping into 9 clusters, *k* = 9.
  As shown in Figure 6, the higher the number of clusters, the higher the explained variance of the rivet installation data. Considering that riveted samples were obtained in 9 groups (see Table 2), and that higher values of k do not significantly increase the amount of variance explained, an analysis with *k* = 9 was performed.

## 4. Analysis of Results and Discussion

### 4.1. Clustering Considering k = 2 Clusters

**Figure 7:**
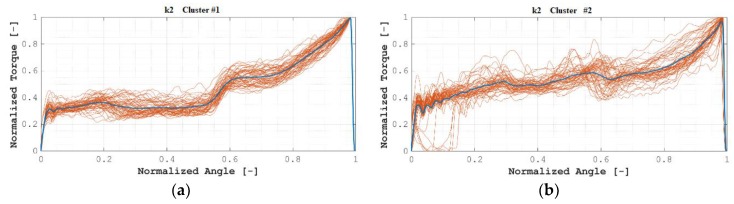
(*k* = 2) (**a**) Cluster #1; (**b**) Cluster #2.

Clustering into *k* = 2 clusters lead to the clusters as shown in Figure 7, where all normalized angle/normalized torque plots are overlaid, and where the centroid of each cluster is remarked. Correspondingly, Table 3 lists the distribution of the samples among both clusters.

**Figure 8:**
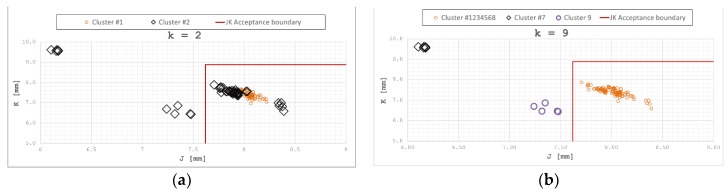
(**a**) (*k* = 2) Distribution of clusters in the *J*–*K* plane; (**b**) (*k* = 9) distribution of clusters in the *J*–*K* plane.

As can be seen in Table 3, all members of Cluster #1 correspond to OK installations, while members of Cluster #2 are either OK or NOK installations. Figure 8a shows this behavior in the *J*–*K* plane for a better understanding.

Whereas members of Cluster #1 are all OK installations, a similar approach of a new installation towards Cluster #1 could lead to a partial method for assessing the quality of the installation: If the new installation is categorized as a member of Cluster #1, then the installation would be considered as OK. Nevertheless, if it were categorized as a member of Cluster #2, then no categorization would be possible.

Thus, clustering with *k* = 2 was considered not valid as a basis for obtaining a classification method of the quality of the installation of blind rivets.

### 4.2. Clustering Considering k = 9 Clusters

Table 4 shows that all members of each cluster correspond to either OK or NOK installations. Their positions in the *J*–*K* plane is showed in Figure 8b, along with *J*_min_ and *K*_max_ limit values, as defined in Reference [16].

Table 4 shows that all members of each cluster correspond to either OK or NOK installations. Their positions in the *J*–*K* plane is shown in Figure 8b, in which Clusters #1, #2, #3, #4, #5, #6, and #8 are shown grouped together for clarity.

The correlation between each cluster and installation quality was thus observed, showing the installation belonging to clusters #1, #2, #3, #4, #5, #6, and #8 as correct, and those of clusters #7 and #9 incorrect. 

### 4.3. Further Analysis of Grip-Thickness Sensitivity

Clustering by the *k*-means method proved its potential to classify OK or NOK installations. Considering that all NOK installations corresponded to either too long or too short rivets (a higher or smaller grip than it should correspond to), a deeper analysis of the capabilities of the method with respect to rivet grip and joint thickness was performed.

A total of 63 sample installations were prepared with progressive varying thickness of the probe from 7.91 mm to 8.24 mm, which corresponded to a 350 grip. In total, 10 of them (16%) were randomly separated as test cases, and the other 53 were used for training the *k*-means clustering with *k* = 3, as shown in Table 5. Table 6 shows the distribution of sample installations among each cluster.

**Figure 9:**
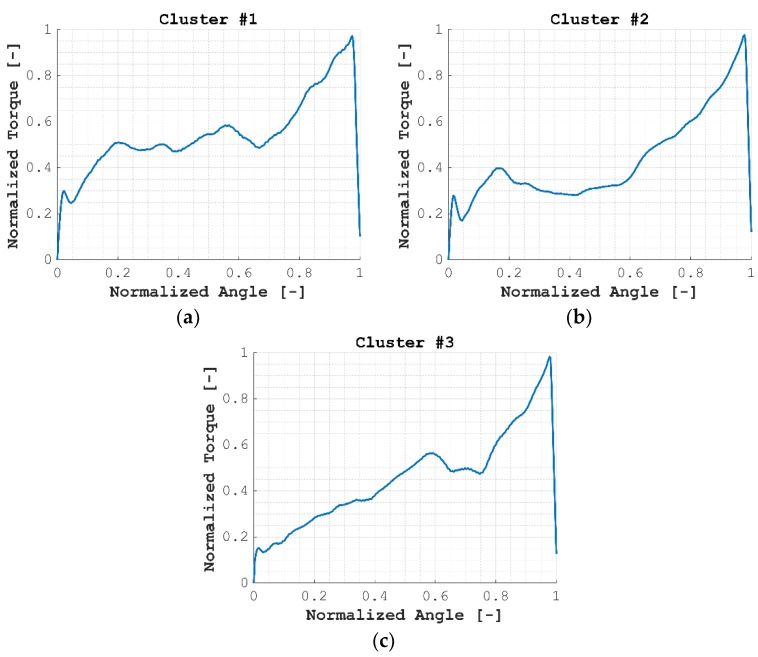
Centroids obtained for varying thickness samples: (**a**) Centroid #1, (**b**) Centroid #2, (**c**) Centroid #3.

The centroid of each cluster is shown in Figure 9, where it was observed how centroids #1 and #2 showed more similarities among them than those with relation to centroid #3 (see for instance the initial states, between normalized angles 0–0.2), while centroid #3 behaved in a more linear manner until the normalized angle, 0.5.

The sensitivity is measured via the capability to correctly classify test rivets. For each test rivet, the Euclidean distance between itself and each of the 3 centroids was computed, and it was classified as belonging to the closest cluster in terms of such distance. Distances are normalized with respect to the minimum value for clarity (a value of 1 corresponds to its assigned cluster) and are shown in Table 7.

Finally, Table 8 shows the confusion matrix of the method on test data, which leads to an accuracy of 90%.

## 5. Conclusions and Future Work

The analysis of a process installation variable, as a fastening torque, has shown its potential for being the basis of a rivet installation quality assessment method. Current research sets the basis for a particularization of the proposed method:

- Varying torque diagrams have been identified, whether the installation was OK or NOK,
- A *k*-means base clustering approach, to automatically and successfully identify patterns and classify them, is presented,
- Clustering manages to separate correct (OK) and incorrect (NOK) installations,
- Experimental results show the potential of the proposed approach in blind fasteners installation, providing a fully-automatic, yet accurate, evaluation system,
- Further tests analyzing the ability of the method with respect to thickness have shown an accuracy of 90%,
- Further tests involving more data must be conducted and the same approach should be tested in other similar industrial processes.

## Figures and Tables

**Table 1:** Typical installation defects in the formed head of blind threaded fasteners (source: Airbus).

Scheme	Installation	Status
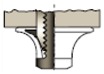	Correct installation	–
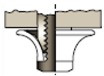	Sleeve not in contact with the structure (loose joint)	Unacceptable
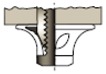	Split or cracked sleeve	Unacceptable
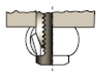	“buckled” sleeve	Unacceptable
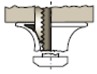	Blind head failure	Unacceptable
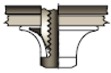	Nut head failure	Unacceptable
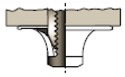	Flared sleeve	Unacceptable if certain limits are not respected

**Table 2:** Description of created samples: 108 total samples, 98 correctly installed (OK), 10 incorrectly installed (NOK).

Reference	Characteristics of Installation, Field of Analysis	Grip	Probe Thickness (mm)	Number of Samples	Installation
HTM01	Nominal installation conditions. Set-up test. No misalignment, 100 N preload, 4.5 bar air pressure	350	8.38	20	OK
HTM02	Misalignment between 10°–20°, out of misalignment tolerances.	350	8.49	38	OK
HTM03_A	Higher grip than specified for the probe’s thickness	500	8.49	5	NOK
HTM03_B	Lower limit of the grip specified for the probe’s thickness was selected	500	12.7	5	OK
HTM03_C	Upper limit of the grip specified for the probe’s thickness was selected.	500	11.5	5	OK
HTM03_D	Lower grip than specified for the probe’s thickness	500	15	5	NOK
HTM04	3× different air pressures used (1.5, 2.5 and 3.5 bar)	500	11.5	9	OK
HTM05	3× different preload values (20, 60, 400, 800 N)	500	11.5	12	OK
HTM06	3× different initial positions of the nut	500	11.5	9	OK

**Table 3:** Distribution of samples among clusters (*k* = 2).

Cluster	Number of Samples
Cluster #1	56
correctly installed	56
Cluster #2	52
correctly installed	42
HTM03_A	5
HTM03_D	5

**Table 4:** Distribution of samples among clusters (*k* = 9).

Cluster	Number of Samples	Comments
Cluster #1	18	HTM01, HTM02
Cluster #2	18	HTM01, HTM02
Cluster #3	22	HTM01, HTM02
Cluster #4	5	HTM03_B
Cluster #5	14	HTM03_C, HTM04, HTM05, HTM06
Cluster #6	10	HTM03_C, HTM04, HTM05, HTM06
Cluster #7	5	HTM03_D
Cluster #8	11	HTM03_C, HTM04, HTM05, HTM06
Cluster #9	5	HTM03_A

**Table 5:** Description of created samples for grip-thickness sensitivity analysis.

Reference	Characteristics of Installation	Number of Samples (Number of Test Samples)	Installation
HTM11	Too short grip (300)	21 (3)	OK
HTM12	Corresponding grip (350)	21 (3)	OK
HTM13	Too long grip (400)	21 (4)	NOK

**Table 6:** Distribution of varying thickness samples among clusters.

Cluster	Number of Samples	From HTM11	From HTM12	From HTM13	Comments	Assigned Installation to Cluster
Cluster #1	11	8	0	3	Mostly too short grips	OK
Cluster #2	28	9	18	1	Mostly correct grip	OK
Cluster #3	14	1	0	13	Mostly too long grips	NOK

**Table 7:** Distribution of varying thickness samples among clusters.

Rivet #	Reference	Installation	Distance to Centroid #1	Distance to Centroid #2	Distance to Centroid #3	Assigned Cluster	Assigned Installation
34	HTM12	OK	3.1	1.0	2.8	2	OK
5	HTM11	OK	2.9	1.0	2.2	2	OK
7	HTM11	OK	1.0	1.4	1.3	1	OK
9	HTM11	OK	1.7	1.0	1.2	2	OK
43	HTM13	NOK	2.8	3.1	1.0	3	NOK
31	HTM12	OK	4.6	1.0	3.4	2	OK
54	HTM13	NOK	1.9	2.0	1.0	3	NOK
59	HTM13	NOK	1.4	2.1	1.0	3	NOK
44	HTM13	NOK	1.0	1.6	1.0	1	OK
37	HTM12	OK	1.4	1.0	1.6	2	OK

**Table 8:** Confusion matrix of the method.

*k*-Means	Installation OK	Installation NOK
Assigned OK	6	1
Assigned NOK	0	3
